# Machine learning model based on dual-layer detector spectral CT radiomics features for differentiating luminal and non-luminal breast cancer

**DOI:** 10.3389/fonc.2026.1739346

**Published:** 2026-02-05

**Authors:** Zhijing Song, Yikun Ma, Zhiyang Dou, Bo Shi

**Affiliations:** 1 Anhui Key Laboratory of Digital Medicine and Intelligent Health, School of Medical Imaging, Bengbu Medical University, Bengbu, China; 2 Department of Radiology, Nanjing Medical University Affiliated Cancer Hospital, Jiangsu Cancer Hospital, Jiangsu Institute of Cancer Research, Nanjing, China

**Keywords:** breast cancer, dual-layer detector spectral CT, machine learning, molecular subtype, radiomics

## Abstract

**Objective:**

This study aims to explore the value of a machine learning (ML) model based on dual-layer detector spectral CT (DLCT) radiomic features in predicting Luminal versus non-Luminal breast cancer (BC).

**Methods:**

A retrospective analysis was conducted on 128 pathologically confirmed BC patients from the Department of Breast Surgery, Jiangsu Cancer Hospital. DLCT chest enhancement images were analyzed, with regions of interest delineated to extract radiomic features. Optimal features were selected through univariate analysis, correlation analysis, and LASSO algorithm, followed by ML model construction.

**Results:**

A total of 1,037 radiomic features were extracted, from which 13 optimal features were selected. Combined with clinical parameters (age, body mass index (BMI), and menopausal status), seven ML models were constructed. Among them, the Gaussian Naive Bayes (GNB) model demonstrated the best performance, achieving an area under the receiver operating characteristic curve (AUC) of 0.778 (95% CI: 0.582–0.974), accuracy of 0.821, sensitivity of 0.833, and specificity of 0.778, outperforming the other six models.

**Conclusions:**

The GNB model demonstrated relatively superior and stable predictive performance in internal testing, suggesting that DLCT radiomics may offer a potential auxiliary tool for distinguishing between Luminal and non-Luminal BC. However, further validation through large-scale multicenter studies is required.

## 1 Introduction

Breast cancer (BC) is one of the most common malignancies in women worldwide, particularly affecting women aged 30 to 45 years (1). It is the second most deadly malignancy affecting women (2), representing a major health concern for female populations. According to the St. Gallen International Breast Cancer Expert Consensus (3), BC can be classified into four subtypes based on four immunohistochemical markers (4). Among these, Luminal A and Luminal B types are categorized as Luminal-type BC, accounting for approximately 60% to 70% of all cases (5), and typically respond well to endocrine therapy (6, 7). Meanwhile, HER2-OE and triple-negative types are classified as non-Luminal BC, often requiring targeted therapy or more intensive treatment regimens, and are associated with poorer prognosis (8–10). Since these two types show significant differences in treatment selection and prognosis (11), accurate preoperative differentiation of subtypes is crucial for developing individualized treatment plans. Currently, BC molecular subtyping primarily relies on histopathological detection methods such as immunohistochemistry and *in situ* hybridization, but these techniques are invasive and prone to sampling bias (12).

In recent years, radiomics has emerged as a research focus for preoperative BC subtyping due to its non-invasive nature and strong reproducibility. Numerous studies have attempted to develop predictive models based on imaging modalities such as magnetic resonance imaging (MRI) and ultrasound (US) (13–16). However, MRI examinations are costly, time-consuming, and susceptible to motion artifacts (17). More importantly, its multi-parameter scanning characteristics affect the stability of radiomic features, thereby compromising model generalizability (18). US demonstrates limited capability in discriminating small lesions (19), while its operator-dependent image acquisition leads to poor reproducibility and biological consistency of extracted radiomic features (20). Therefore, there is an urgent need for more efficient and precise radiomic approaches to optimize preoperative BC subtyping. As an emerging imaging technology, dual-layer detector spectral CT (DLCT) offers high resolution and provides multiple spectral images, thereby expanding radiomics research possibilities (21–23). Preliminary studies have demonstrated DLCT radiomics’ effectiveness in predicting malignant tumors (24, 25), yet its application in BC molecular subtyping remains unexplored.

Therefore, this study aims to innovatively integrate DLCT radiomic features with machine learning (ML) algorithms to construct a non-invasive discrimination model for Luminal versus non-Luminal BC, exploring the potential of DLCT radiomics for precise BC subtyping.

## 2 Methods

### 2.1 Patients

This retrospective study analyzed BC patients treated at the Department of Breast Surgery, Jiangsu Cancer Hospital from October 2021 to July 2024. Inclusion criteria comprised: (1) Confirmed as BC through histopathological examination; (2) Preoperative contrast-enhanced DLCT chest examination. Exclusion criteria eliminated patients with: (1) Prior surgical/radiation/chemotherapy treatments (n=4); (2) >1 week interval between imaging and pathological confirmation (n=3); (3) Suboptimal image quality hampers ROI delineation (n=3); (4) Incomplete clinicopathological records (n=5). After applying these selection criteria, 128 patients qualified for final analysis.

### 2.2 Clinical and histopathological analysis

We analyzed clinical and pathological data from all patients, including age, BMI, menopausal status, and immunohistochemistry (IHC) results. Based on the 2013 St. Gallen International Breast Cancer Expert Consensus (3), patients were classified into four molecular subtypes using four IHC markers: estrogen receptor (ER), progesterone receptor (PR), human epidermal growth factor receptor 2 (HER2), and Ki-67 proliferation index. The classification criteria were: (1) Luminal A: ER(+) or PR(+), HER2(-), Ki-67 <20%; (2) Luminal B: ①:minal,cationm ER(+) or PR(+), HER2(-), Ki-67 ≥i-67 ②i-67-),cationm ER(+) or PR(+), HER2(+), any Ki-67; (3) HER2-OE: ER(-), PR(-), HER2(+), any Ki-67; (4) TNBC: ER(-), PR(-), HER2(-), any Ki-67. For subsequent analysis, Luminal A and B subtypes were grouped as Luminal-type BC, while HER2-OE and TNBC were classified as non-Luminal BC.

### 2.3 DLCT image acquisition

All patients underwent preoperative contrast-enhanced DLCT chest examinations using the IQon spectral CT scanner (Philips Healthcare, Best, The Netherlands). Patients were positioned supine with a scanning range extending from the lung apex to the costophrenic angle level. For contrast-enhanced imaging, a non-ionic iodinated contrast agent (ioversol, 350 mg iodine/mL, Hengrui Pharmaceuticals, Lianyungang, China) was administered intravenously at an injection rate of 2.5-3.0 mL/s, followed by a 20 mL saline flush at 2.5 mL/s. Post-injection scanning was initiated after a 50-second delay. The scanning parameters were as follows: tube voltage 120 kVp, automatic tube current modulation, detector configuration 64 × 0.625 mm, pitch 0.900, rotation time 0.50 s, matrix size 512 × 512, field of view 372 mm, scan slice thickness 5 mm, and reconstruction slice thickness 1 mm.

### 2.4 Image segmentation and radiomics feature extraction

This study performed radiomics feature extraction based on 55 keV images. Previous research has demonstrated that images at this keV level exhibit good image quality and an optimal contrast-to-noise ratio (26, 27). Two experienced radiologists, blinded to clinical and pathological findings, used 3D-Slicer software to manually delineate regions of interest (ROI) slice-by-slice along lesion contours on 55 keV monochromatic images. Any discrepancies in ROI delineation were resolved through consensus. After ROI delineation, images were resampled to 1×1×1 mm voxels, and feature extraction was performed using the PyRadiomics package. Extracted features included: (1) Original features: 14 shape features, 18 first-order statistical features, and 75 texture features (including 24 from gray-level co-occurrence matrix (GLCM), 16 from gray-level size zone matrix (GLSZM), 16 from gray-level run length matrix (GLRLM), 5 from neighboring gray-tone difference matrix (NGTDM), and 14 from gray-level dependence matrix (GLDM); (2) Transformed features obtained after image filtering using wavelet transform (combining high-pass and low-pass filters in three directions) and Laplacian of Gaussian filters (3, 5).

### 2.5 Radiomics feature selection and model construction

The dataset was randomly divided into training and test sets at a ratio of 7:3. Z-score normalization was applied exclusively to the training set data, and the Synthetic Minority Oversampling Technique (SMOTE) was employed to achieve a 1:1 ratio between Luminal and non-Luminal samples. The working principle of this method involves randomly generating a certain number of new samples along the line segments between existing minority class samples. This approach not only addresses the class imbalance issue but also effectively reduces the risk of model overfitting, thereby enhancing the model’s generalization performance to some extent (28, 29). In the training set, univariate analysis was performed on the data, followed by correlation testing to remove redundant features, retaining only one feature when r > 0.7. The Lasso algorithm (with 5-fold cross-validation) was then applied to eliminate features with coefficients of zero. This method can effectively handle multicollinearity among features and offers higher computational efficiency compared to iterative wrapper methods, such as Recursive Feature Elimination. Moreover, the results of the Lasso algorithm (with 5-fold cross-validation) represent a subset of the original feature space (30). In contrast to Principal Component Analysis, this approach facilitates the direct presentation of the radiomics features that drive the model’s decision-making. Finally, the selected features were ranked by importance based on the model coefficients to identify stable and key features. The test set remained completely isolated throughout the entire feature selection process and was used solely for final performance evaluation. After balancing the data in the training set using the SMOTE method, a 10-fold cross-validation approach was employed, dynamically partitioning the data into training and validation sets. The model was trained on the training and validation sets and ultimately evaluated on the test set.

Seven ML algorithms were used to construct radiomics models: logistic regression (LR), extreme gradient boosting (XGBoost), light gradient boosting machine (LightGBM), adaptive boosting (AdaBoost), random forest (RF), Gaussian naive Bayes (GNB), and support vector machine (SVM). Model performance was evaluated using the receiver operating characteristic (ROC) curve, the area under the ROC curve (AUC) and its 95% confidence interval (95% CI), accuracy, sensitivity, and specificity. The models were assessed through 10-fold cross-validation and further validated using test set data. For the calculation of classification performance metrics such as sensitivity and specificity, the predicted probabilities from the models were binarized using the default threshold of 0.5: probabilities ≥ 0.5 were assigned as positive, and probabilities < 0.5 were assigned as negative. For the best-performing model, confusion matrix plots and learning curve plots were generated, and visual interpretation was conducted using Shapley additive explanations (SHAP) explainability analysis method.The above modeling process was based on Python programming language (version 3.11.4). All models were trained using the default parameters of their standard implementation libraries. The XGBoost model was implemented using xgboost=2.0.1, the LightGBM model was implemented using lightgbm=3.2.1, and the other models were implemented using scikit-learn=1.1.3.

### 2.6 Statistical analysis

Statistical analyses were performed using SPSS Statistics 27.0 (IBM Corp, Chicago, Illinois, United States of America). The Shapiro-Wilk test was used to assess data normality. Normally distributed continuous data were expressed as mean ± standard deviation (SD) and compared using independent samples t-tests. Non-normally distributed continuous data were expressed as [M50 (P25, P75)] and compared using Mann-Whitney *U* tests. Categorical data were expressed as [n(%)] and compared using chi-square tests. A *p* value < 0.05 was considered statistically significant.

## 3 Results

### 3.1 Participant characteristics

This study ultimately included 128 BC patients (all female), comprising 33 cases (25.8%) of non-Luminal type with mean age 56.4 ± 10.4 years and mean BMI 24.2 ± 3.3 kg/m², and 95 cases (74.2%) of Luminal type with mean age 53.9 ± 11.7 years and mean BMI 24.6 ± 3.1 kg/m². There were 40 premenopausal patients (31.3%) and 88 postmenopausal patients (68.7%). **Table 1** presents the patients’ baseline data and clinical characteristics. No statistically significant differences were observed between the two groups regarding age, BMI, or menopausal status.

**Table 1:** Comparison of clinical data of all patients.

Characteristics	Total (N = 128)	Non-Luminal (N = 33)	Luminal (N = 95)	*P* value
Age (years, mean ± SD)	54.6 ± 11.4	56.4 ± 10.4	53.9 ± 11.7	0.256
BMI (kg/m^2^, mean ± SD)	24.5 ± 3.1	24.2 ± 3.3	24.6 ± 3.1	0.542
Menopause				0.723
No	40 (31.3%)	9 (27.3%)	31 (32.6%)	
Yes	88 (68.7%)	24 (72.7%)	64 (67.4%)	

### 3.2 Radiomics feature extraction and selection

**Figure 1:**
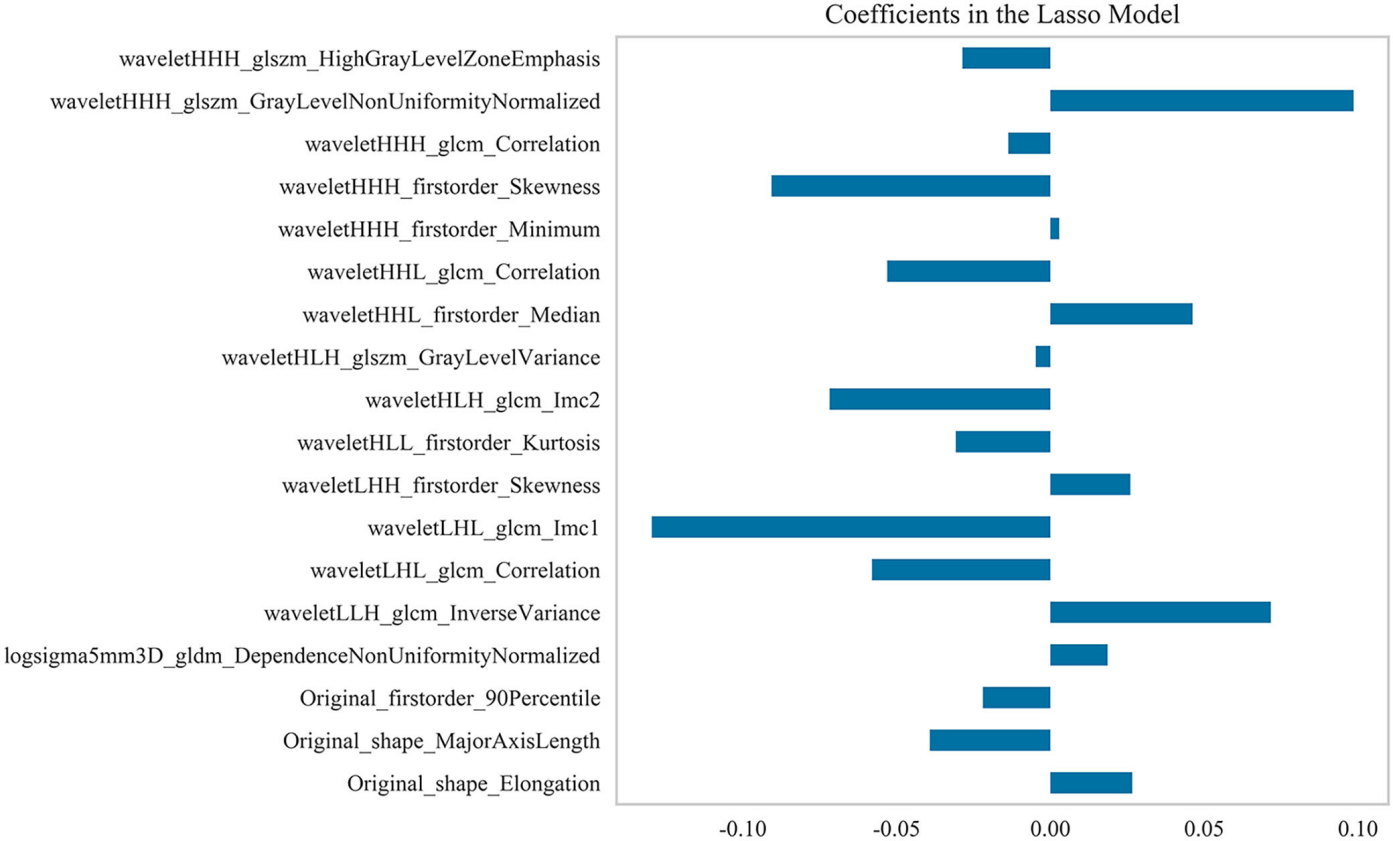
The names and coefficient plot of the radiomics features selected by the Lasso algorithm (with 5-fold cross-validation).

**Figure 2:**
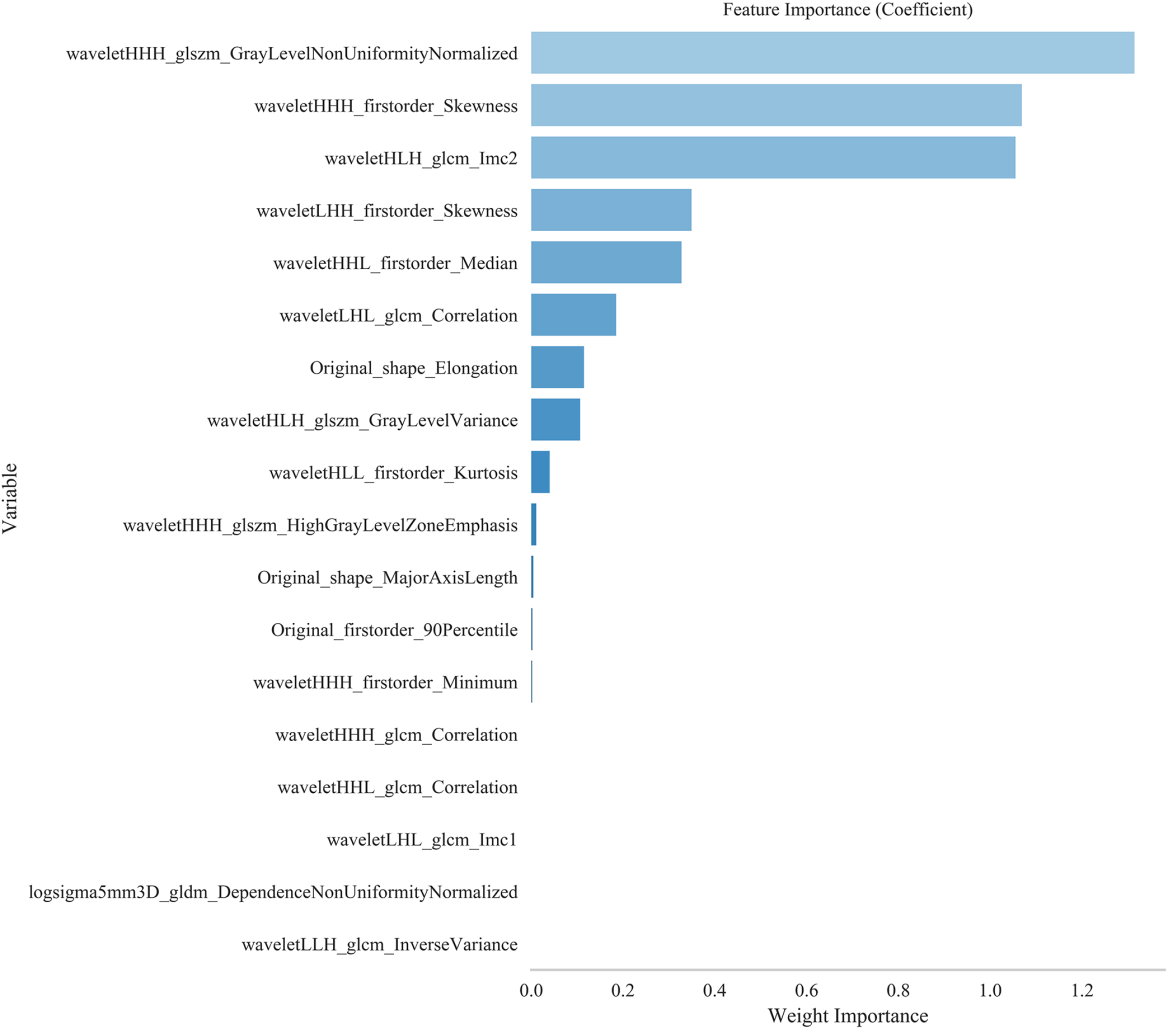
The importance ranking chart of 18 features. The features are arranged from top to bottom based on their importance, and the longer the bar length, the more significant the feature.

Based on 55 keV contrast-enhanced DLCT chest images from 128 patients, a total of 1,037 radiomic features were extracted from the region of interest delineated within each patient’s lesion. According to a 7:3 ratio, the dataset was randomly divided into a training set and a test set, with 89 samples in the training set and 39 samples in the test set. After applying SMOTE, the training set comprised 130 samples. In each fold of the 10-fold cross-validation, 117 cases were used for training, and 13 cases were used for validation. Through univariate analysis and correlation analysis, 39 features were initially selected, which were further reduced to 18 features using the Lasso algorithm. The optimal regularization parameter was 0.026. **Figure 1** displays the names and coefficient plot of the selected radiomics features. **Figure 2** displays the importance ranking of these 18 features based on model coefficient analysis. Ultimately, the top 13 features with low correlation but high discriminative power were selected for subsequent modeling analysis.

### 3.3 Diagnostic performance of the seven models

**Figure 3:**
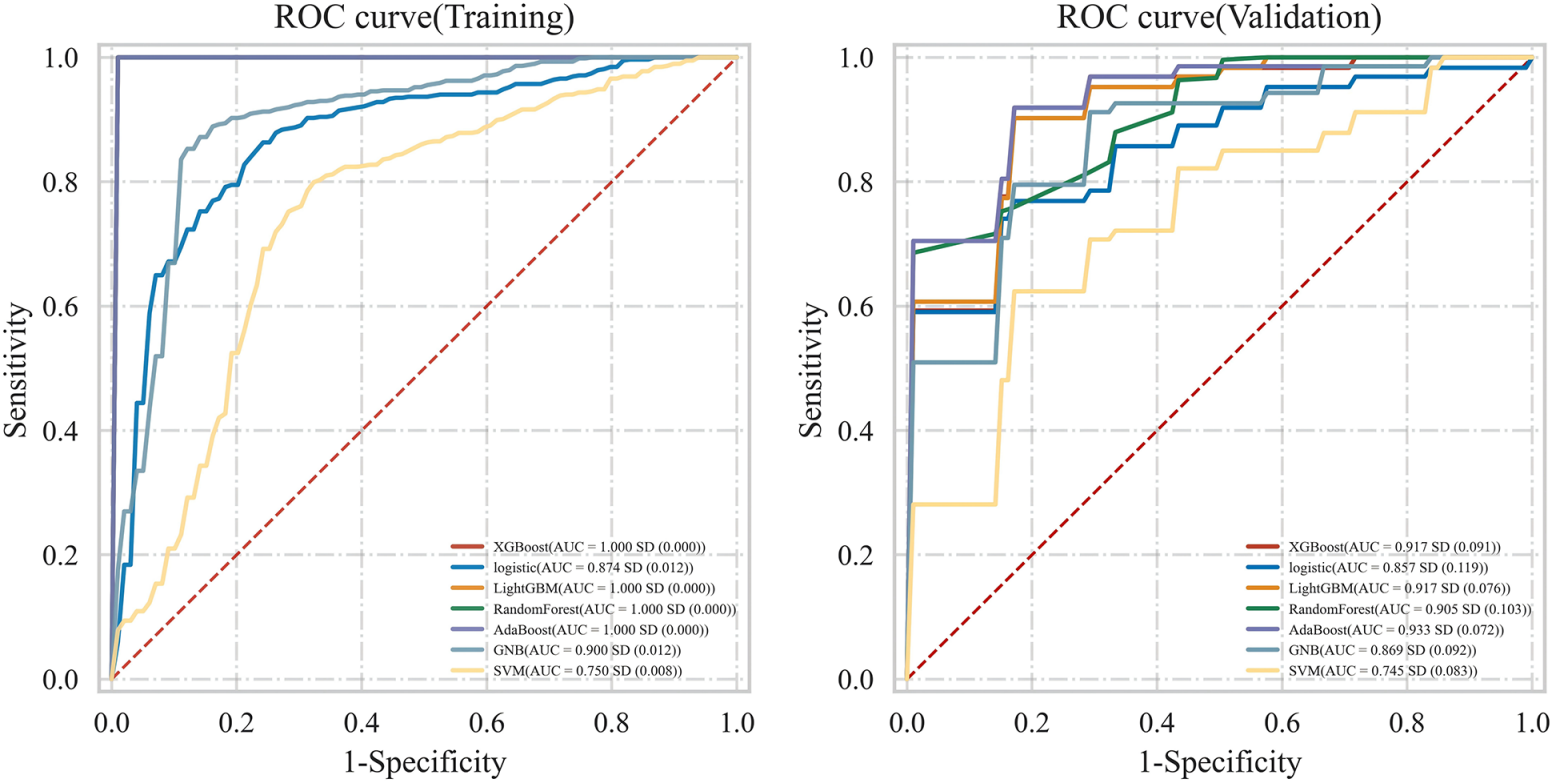
The ROC curves of 7 models on the training set and validation set.

**Figure 4:**
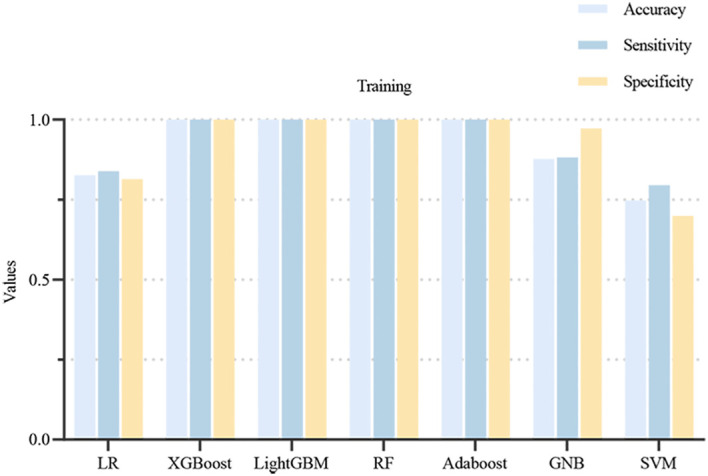
Performance metrics of the 7 models from 10-fold cross-validation in the training set. The light blue bar represents the accuracy of the model, the dark blue bar represents the sensitivity of the model, and the yellow bar represents the specificity of the model.

**Figure 5:**
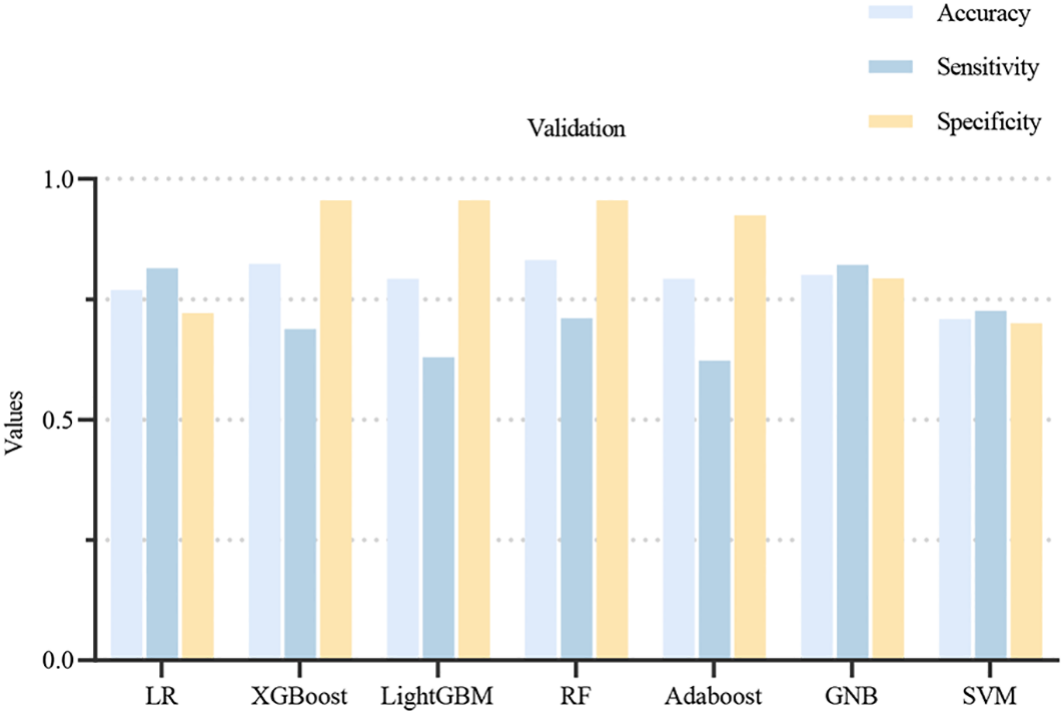
Performance metrics of the 7 models from 10-fold cross-validation in the validation set. The light blue bar represents the accuracy of the model, the dark blue bar represents the sensitivity of the model, and the yellow bar represents the specificity of the model.

**Figure 6:**
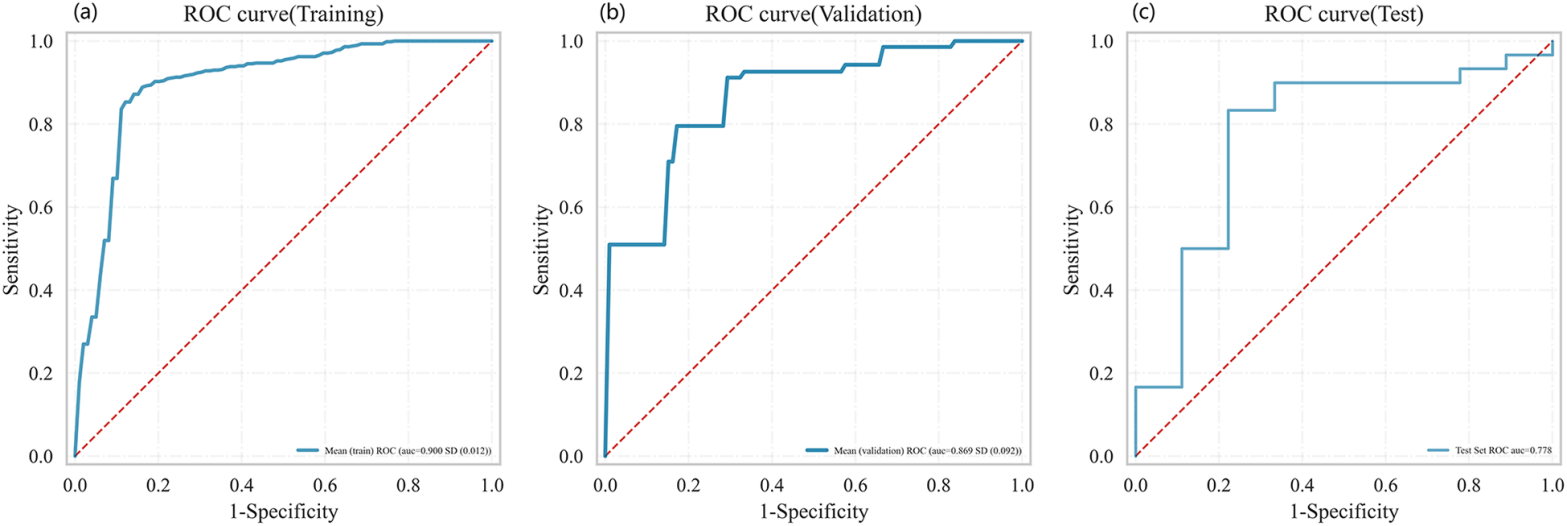
The ROC of the GNB model. **(a–c)** represent the training set, validation set and test set respectively.

**Figure 7:**
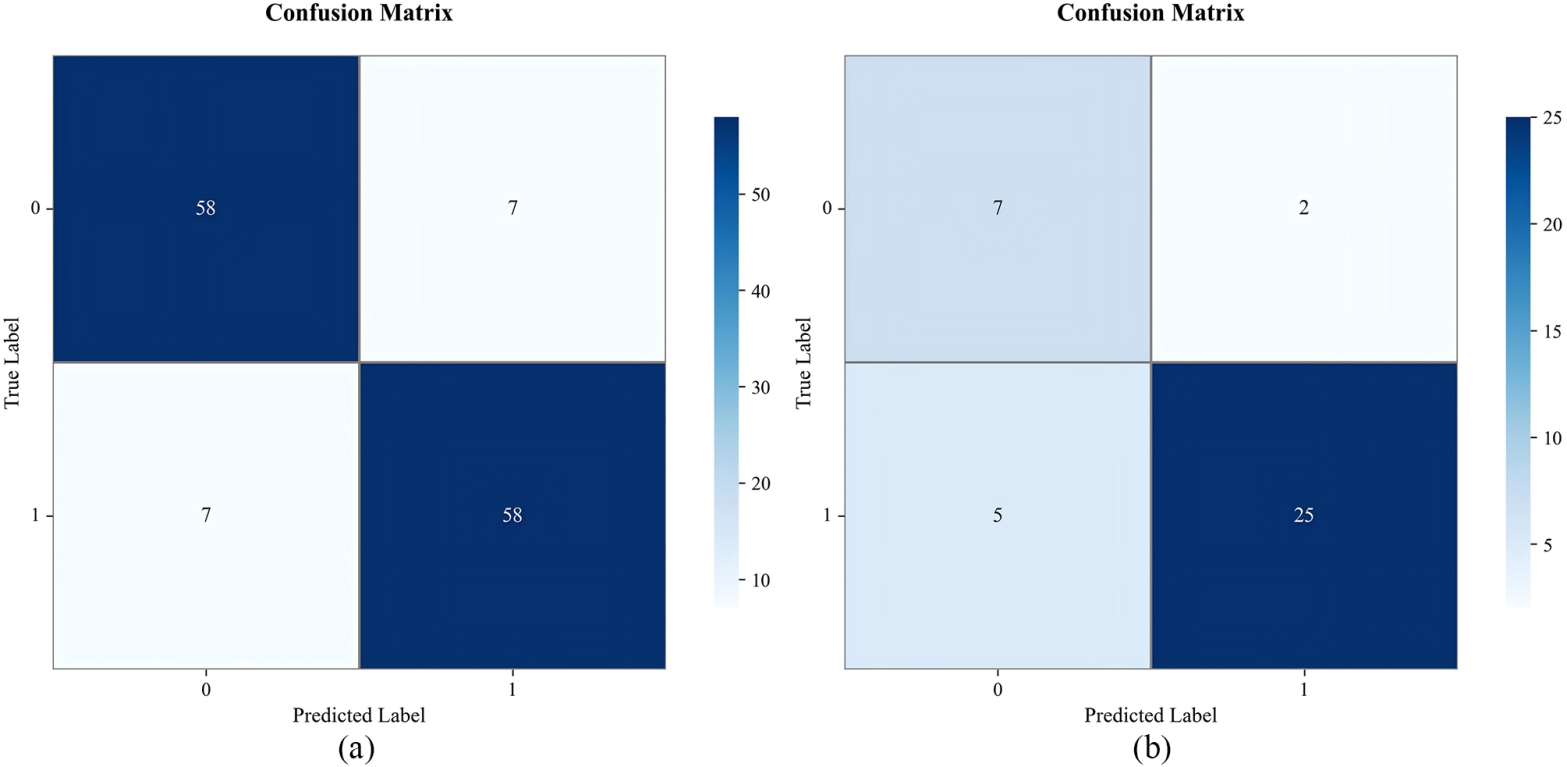
Confusion matrix plots of the GNB model for the training set and test set. **(a)** corresponds to the training set, and **(b)** corresponds to the test set.

**Figure 8:**
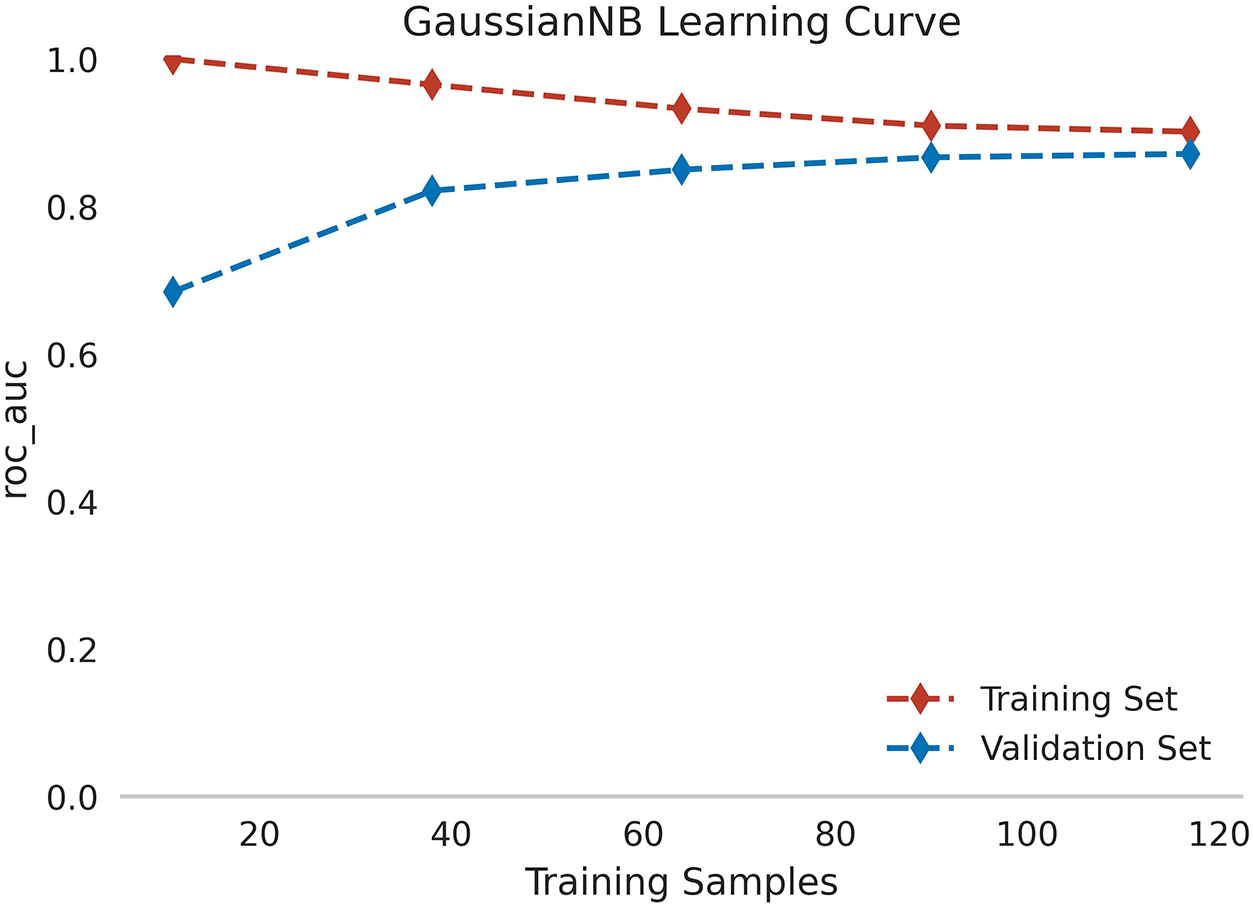
Learning curves of the GNB model on the training set and validation set.

Using 13 radiomic features and 3 clinical features, we constructed prediction models with seven ML algorithms. **Figure 3** shows the ROC curves of these seven models in both the training and validation sets. **Figures 4**, **5** present the performance metrics of the seven models in the training and validation sets using 10-fold cross-validation. **Table 2** lists the performance metrics of the seven models in the test set. As can be seen from **Figures 4**, **5**; **Table 2**, although the XGBoost, LightGBM, AdaBoost and RF models showed higher AUC values in the training and validation sets, their performance in the test set was unsatisfactory, suggesting possible overfitting. The GNB model achieved AUC values of 0.900 and 0.869 in the training and validation sets, respectively. In the test set, the model attained an AUC of 0.778 (95% CI: 0.582–0.974), accuracy of 0.821, sensitivity of 0.833, and specificity of 0.778, outperforming the other six models. From the sensitivity and specificity of the GNB model, it can be observed that both values are comparable, showing no severe performance bias caused by the original sample imbalance. The results indicate that the GNB model had the best predictive performance. **Figure 6** displays the ROC curves of the GNB model across the training, validation, and test sets. **Figure 7** shows the confusion matrix of the GNB model. **Figure 8**. Learning curves of the GNB model on the training set and validation set.

**Table 2:** Performance indicators of 7 models in the test set.

Models	AUC (95% CI)	Accuracy	Sensitivity	Specificity
LR	0.578 (0.387 - 0.769)	0.487	0.567	0.222
XGBoost	0.585 (0.363 - 0.808)	0.513	0.533	0.444
LightGBM	0.530 (0.302 - 0.757)	0.538	0.567	0.444
RF	0.565 (0.347 - 0.783)	0.538	0.533	0.556
Adaboost	0.552 (0.349 - 0.755)	0.564	0.633	0.333
GNB	0.778 (0.582 - 0.974)	0.821	0.833	0.778
SVM	0.667 (0.469 - 0.864)	0.667	0.667	0.667

### 3.4 Model interpretation

**Figure 9:**
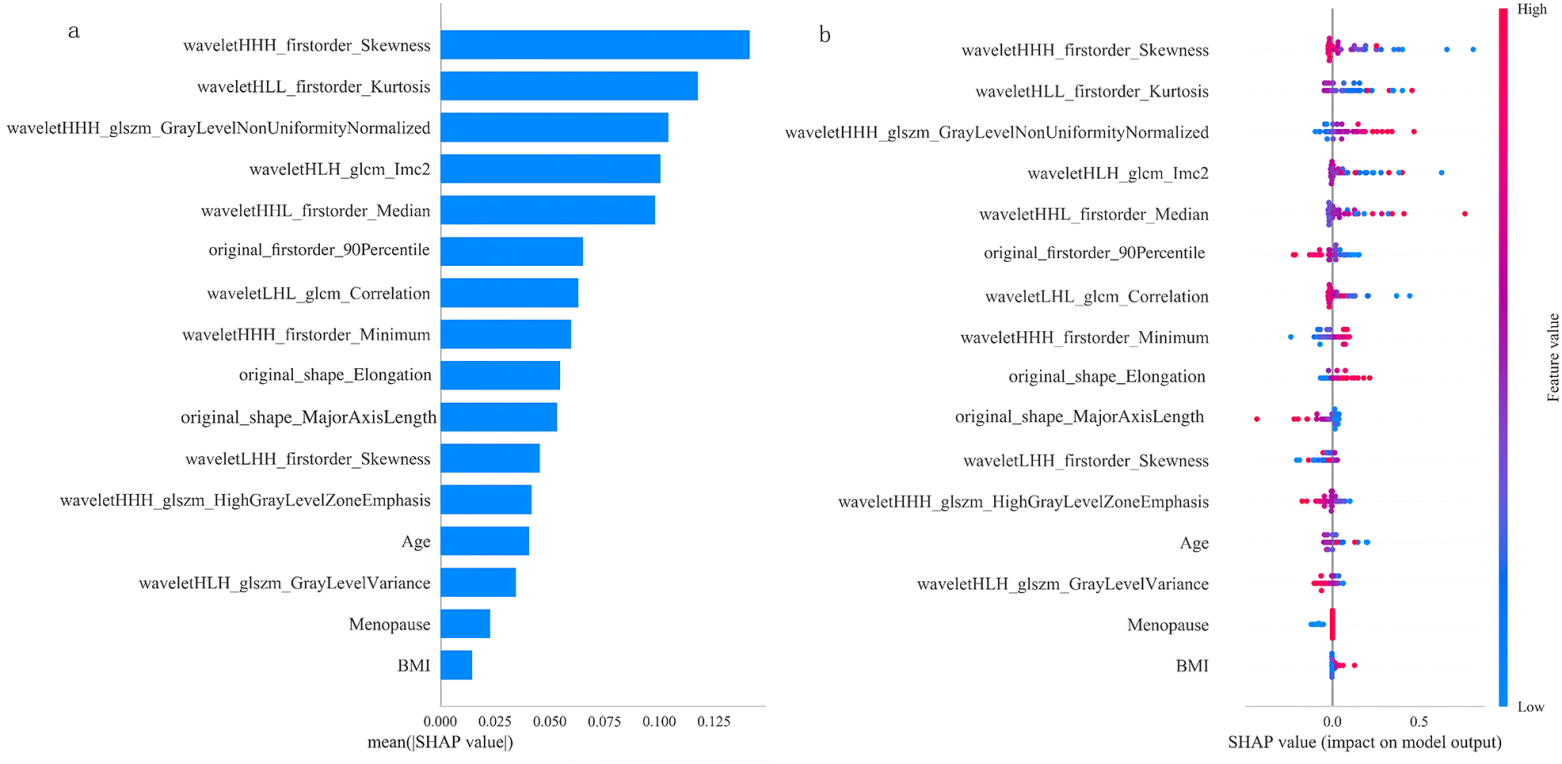
SHAP bar plot and summary plot of the GNB model. **(a)** SHAP bar plot of the GNB model. The vertical axis lists various features, sorted by their average impact on the model’s predictions with the most important features positioned at the top. The horizontal axis represents the absolute value of the average SHAP value for each feature’s contribution to the model’s predictions, reflecting the feature’s importance. **(b)** SHAP summary plot of the GNB model. The y-axis displays the features in the model, ranked by their contribution to the model’s predictions, with the most important features positioned at the top. The color of each data point represents feature values—red indicates higher values, while blue indicates lower values. The x-axis represents the SHAP value for each data point, where positive values contribute to predicting a positive outcome, and negative values contribute to predicting a negative outcome.

Visual analysis was performed on the best-performing GNB model using SHAP method. **Figure 9a** displays the contribution values of all 16 features incorporated in the GNB model. **Figure 9b** presents the SHAP summary plot for the Gaussian Naive Bayes (GNB) model, with features ranked vertically by their mean absolute SHAP values in descending order. Higher-positioned features demonstrate stronger predictive importance for discriminating between Luminal and non-Luminal subtypes.

## 4 Discussion

This study is the first to propose a ML model based on DLCT chest enhancement imaging radiomic features for distinguishing Luminal from non-Luminal BC. The results demonstrate that the GNB model combining 13 radiomic features and 3 clinical features exhibits good predictive performance (AUC = 0.778). These findings provide valuable reference for early diagnosis and precision treatment of Luminal and non-Luminal BC, offering new imaging evidence for future subtyping research.

Recent years have seen increasing research focus on developing non-invasive methods for early differentiation between Luminal and non-Luminal BC. Studies by Xu et al. (31), Feng et al. (32), and Wang et al. (33) developed models using MRI radiomic features and functional parameters, with AUC values of 0.830, 0.879, and 0.830 respectively. Liu et al. (34) developed a model using US radiomic features with an AUC of 0.752. In terms of model performance, MRI-based models outperformed US-based ones. Umutlu et al. (35) created a model using PET/MRI radiomic features with an AUC of 0.950, though without further test set validation and with higher PET/MRI costs. Liu et al. (36) developed a predictive model based on DLCT quantitative parameters with an AUC of 0.754, performing less well than our DLCT radiomics-based model. Our GNB model outperformed US radiomics-based and DLCT quantitative parameter-based models, though showed lower performance than MRI radiomics, functional parameter, and PET/MRI radiomics-based models. However, compared to MRI and PET/MRI examinations, DLCT offers higher examination efficiency and lower cost. Additionally, unlike the prone position typically used in MRI, DLCT’s supine position matches surgical positioning, minimizing potential image and lesion deviations caused by posture changes, while simultaneously evaluating skin, chest wall, internal mammary lymph nodes, bilateral axillary lymph nodes, and supraclavicular lymph nodes (37).

Among the seven ML models constructed based on the same DLCT radiomics features, **Figures 4**, **5**; **Table 2** reveal that the XGBoost, LightGBM, AdaBoost, and RF models exhibit outstanding predictive performance on both the training and validation sets (AUC = 1.000 on the training set, and AUC > 0.900 on the validation set). However, their performance significantly declines on the independent test set (AUC < 0.600). This marked performance discrepancy strongly suggests the presence of overfitting in these models. We attribute the occurrence of overfitting primarily to the high-dimensional, small-sample challenge faced in this study. A total of 1,037 features were extracted from each patient’s images, while the initial training set comprised only 89 samples, expanding to 130 after SMOTE balancing. In such a scenario where the feature dimension substantially exceeds the sample size, models are highly prone to capturing noise and spurious correlations in the training data, leading to diminished generalization capability. To mitigate overfitting, multiple safeguards were incorporated into the study pipeline, such as SMOTE, LASSO, cross-validation, and the strict establishment of an independent test set. Although overfitting was observed in some complex models, the GNB model we ultimately selected demonstrated the smallest performance gap across the training, validation, and test sets, with AUC values of 0.900, 0.869, and 0.778, respectively. Although the GNB model assumes strong independence among features, multiple previous radiomics modeling studies have reported that the GNB model demonstrated the best performance in their research (38, 39). We hypothesize that in high-dimensional, small-sample scenarios, the strong independence assumption of the GNB model can, to some extent, prevent the model from fitting noise and complex feature interactions in high-dimensional data, thereby enhancing its generalization capability. In contrast, more flexible models such as RF and XGBoost are more prone to overfitting. The robust generalization performance of the GNB model is further corroborated by the specific classification patterns revealed in its confusion matrix. As shown in **Figure 7**, the model correctly identified 25 cases of Luminal BC (true positives) and 7 cases of non-Luminal BC (true negatives) in the test set (n=39), achieving an overall accuracy of 84.6%. The model’s errors exhibit a clear asymmetry, with false negatives being the primary source of error, while false positives are relatively fewer. Future feature engineering efforts could focus on these misclassified cases.

Radiomics converts medical images into quantitative, objective features to non-invasively explore tumor heterogeneity and characteristics (16). Existing research has demonstrated radiomics’ potential for non-invasive BC subtyping, though most studies utilized MRI or US. Feng et al. (32) combined clinical factors with intratumoral subregion MRI radiomic features to develop a nomogram model (AUC = 0.830), while Wu et al. (40) created a nomogram based on ultrasound radiomic features (AUC = 0.767). Previous CT-based investigations such as the work of Wang et al. (41), who created a radiomic model distinguishing Luminal from non-Luminal BC (AUC = 0.757) using CT features. To our knowledge, no prior studies have utilized DLCT radiomic features to differentiate between Luminal type and non-Luminal type BC. Our DLCT radiomics-based GNB model achieved test set AUC, accuracy, sensitivity and specificity of 0.778 (95% CI: 0.582–0.974), 0.821, 0.833 and 0.778 respectively, demonstrating good performance. Among the 13 radiomic features ultimately selected for modeling, three were shape features and first-order statistics from the original images: ‘original_shape_MajorAxisLength’ representing the longest axis, ‘original_shape_Elongation’ indicating the ratio of the shortest to longest axis, and ‘original_firstorder_90Percentile’ denoting the 90th percentile value. The other ten features, accounting for the largest proportion, were all wavelet-transformed first-order statistical and texture features. Bian et al. (42) found wavelet features played important roles in their multiparametric MRI-based model predicting HER2-low BC; Yang et al. (43) demonstrated strong correlations between wavelet features and neoadjuvant chemotherapy response; Zhou et al. (44) showed wavelet features’ good performance in evaluating neoadjuvant chemoradiotherapy for BC patients. These findings suggest wavelet features may have predictive value in BC, consistent with our results. From **Figure 9a**, it can be observed that the top five most contributing radiomics features in the GNB model are waveletHHH_firstorder_Skewness, waveletHLL_firstorder_Kurtosis, waveletHHH_glszm_GrayLevelNonUniformityNormalized, waveletHLH_glcm_Imc2, and waveletHHL_firstorder_Median. The wavelet-HHH-firstorder-Skewness measures the asymmetry of the CT value distribution in the tumor region. **Figure 9b** shows that the lower values of this feature are concentrated in the right high SHAP value region. The lower the skewness value, the more the model tends to classify the tumor as the Luminal type. In imaging, lower skewness indicates a more symmetric CT value distribution. We speculate that this may reflect a relatively homogeneous microenvironment in this type of tumor, lacking significant microcalcifications or micro-necrotic areas. Conversely, high skewness may be associated with heterogeneous components in non-Luminal types. Wavelet-HLL-firstorder-Kurtosis describes the kurtosis of the CT value distribution. As observed in **Figure 9b**, the lower the kurtosis value, the more the model tends to classify the tumor as Luminal type. Low kurtosis indicates a broad, dispersed distribution of voxel CT values. In contrast, high kurtosis typically signifies that voxel values are highly concentrated within a narrow range, which may correspond to highly homogeneous areas on imaging, such as large necrotic or liquefied regions or abnormally uniform areas of marked enhancement. These patterns may be more closely associated with certain features of non-Luminal types (45). Wavelet-HHH-glszm-GrayLevelNonUniformityNormalized quantifies the spatial dominance of different density regions in high-frequency texture details. **Figure 9b** shows that the higher its feature value, the greater its contribution to predicting the Luminal type. This suggests that, at the high-frequency texture scale, the microstructure of Luminal BC may be spatially dominated by a few highly homogeneous tissue components. On imaging, this may manifest as dominant regions composed of large, relatively uniform glandular parenchyma or stromal components. Wavelet-HLH-glcm-Imc2 evaluates the complexity and regularity of pixel gray-level co-occurrence patterns. **Figure 9b** shows that the lower its feature value, the greater its contribution to predicting the Luminal type. We hypothesize that the relatively regular structure of Luminal BC results in a low Imc2 value at the algorithmic level. Conversely, a high Imc2 may correspond to extreme homogenization of large-scale structures, such as extensive necrosis or abnormally uniform areas of enhancement, which are common features of non-Luminal BC. It is noteworthy that this study found that higher values of the wavelet-HHL-firstorder_Median feature (indicative of higher enhancement) positively contribute to predicting the Luminal type. This appears to contradict some conventional imaging views that non-Luminal types have richer blood supply (46). We propose that this discrepancy may arise from the following reasons: this study evaluates the median CT value in a specific frequency subband (HHL) after wavelet transformation, rather than the average enhancement of the entire tumor on the original images. The enhancement patterns observed at this specific texture scale may differ biologically from overall enhancement. Radiomics features capture spatial distribution and texture patterns. A high Median value may more strongly reflect the concentration and consistency of enhancement distribution at the HHL scale, rather than merely the peak enhancement intensity. Luminal BC may exhibit more uniform and consistent overall enhancement, whereas non-Luminal BC may display heterogeneous, focal marked enhancement (47).

The GNB model based on DLCT radiomics developed in this study provides proof-of-concept for a rapid, objective, and non-invasive tool for preoperative BC subtyping. We envision the following clinical integration pathway: the model is designed to serve as an auxiliary diagnostic tool, integrated into the post-processing stage of BC imaging examinations. After patients undergo chest contrast-enhanced DLCT scanning, radiologists or technicians can invoke this model on a PACS workstation or a dedicated radiomics analysis platform. By inputting the 55 keV images and performing delineation, the model will automatically extract radiomic features from the ROI and execute the prediction algorithm. Within seconds, the system will generate a structured report containing predicted probability values, confidence intervals, and visualizations of key discriminative features, among other information. The results can serve as an auxiliary reference for radiologists, reducing subjective variability in diagnosis. Moreover, prior to the availability of pathological results, it can provide clinicians with preliminary insights into molecular subtype tendencies, facilitating earlier planning for subsequent treatment strategy discussions.

This study has several limitations. First, the relatively limited sample size may affect the statistical power and stability of the model. Second, all data were derived from a single center, where patient population characteristics, imaging acquisition equipment, and protocols are relatively uniform. This may limit the generalizability of the model developed in this study to other institutions, different equipment, or diverse patient populations. Following this study, we plan to design a prospective study to conduct real-time validation with consecutively enrolled patients at our institution in the future, and actively seek multi-center collaborations. Third, despite the use of the SMOTE algorithm for data balancing, the imbalance in the number of Luminal-type and non-Luminal-type patients may have affected model performance. Future studies should continue to collect cases with an emphasis on balancing patient ratios, and include comparative experiments with and without SMOTE as a key component of the research. Fourth, this study employed manual ROI segmentation. Although strict standardized protocols were followed to ensure quality, the absence of a multi-observer consistency test may somewhat affect the reproducibility of the method. In subsequent research, we will prioritize the adoption of semi-automatic segmentation algorithms or organize multi-center observer agreement studies to further enhance reproducibility. Fifth, this study did not systematically evaluate the impact of variations in imaging acquisition and reconstruction parameters on the stability of radiomic features. Future research must incorporate rigorous testing of feature stability to identify robust features that are insensitive to technical parameters.

## 5 Conclusion

Based on DLCT radiomic features, this study preliminarily explored and constructed seven ML models. Among these, the GNB model demonstrated relatively superior and stable predictive performance in internal testing. The findings suggest that DLCT radiomics may offer a potential auxiliary tool for distinguishing between Luminal and non-Luminal BC, thereby potentially aiding in early diagnosis and preliminary discussions on treatment strategies. This study provides preliminary evidence and hypotheses for this field, but its clinical translation prospects urgently require further validation and advancement through large-scale, prospective, multicenter studies in the future.

## Data Availability

The datasets generated during and/or analyzed during the current study are available from the corresponding author on reasonable request. Requests to access the datasets should be directed to Bo Shi, shibo@bbmu.edu.cn.
